# An Online Documentary Film to Motivate Quit Attempts Among Smokers in the General Population (4Weeks2Freedom): A Randomized Controlled Trial

**DOI:** 10.1093/ntr/ntv161

**Published:** 2015-07-27

**Authors:** Jamie Brown, Susan Michie, Matthew Walmsley, Robert West

**Affiliations:** 1 ^1^ Department of Clinical, Educational and Health Psychology, University College London, London, United Kingdom;; 2 ^2^ Cancer Research UK Health Behaviour Research Centre, Department of Epidemiology and Public Health, University College London, London, United Kingdom;; 3 ^3^ National Centre for Smoking Cessation and Training, London, United Kingdom;; 4 ^4^ Public Health England, London, United Kingdom

## Abstract

**Introduction:**

Online motivational films to promote quit attempts could encourage large numbers of smokers to stop at low unit cost. We evaluated an online film documenting the experiences of smokers who recorded the first month of their successful attempts to quit (4Weeks2Freedom). The film was designed to boost motivation and self-efficacy and provide role-models to promote ex-smoker identities.

**Methods:**

This was a randomized trial with individual assignment to a no-intervention control (*n* = 1016), an informational film (*n* = 1004), or 4Weeks2Freedom (*n* = 999). The development of 4Weeks2Freedom was informed by PRIME theory and focus-group testing with smokers. The 90-minute film was available online to view in one sitting or as chapters over 4 weeks to coincide with the progress of an attempt. The primary outcome was a quit attempt in the 4 weeks between assignment and study endpoint by intent-to-treat.

**Results:**

Participants smoked a mean of 13 cigarettes per day and 31% reported not wanting to stop. At follow-up, 55% reported viewing the informational control film and 56% viewing 4Weeks2Freedom. There was no detectable effect compared with the no-intervention control (*OR* = 0.99, 95% CI = 0.81 to 1.21, 24.3% vs. 24.5%) or informational control film (*OR* = 0.99, 95% CI = 0.80 to 1.21, 24.3% vs. 24.6%). Calculation of Bayes factors ruled out insensitive data and indicated the intervention was no more effective than either the no-intervention control (Bayes factor = 0.20) or informational control film (Bayes factor = 0.27). The pattern of results was unchanged in sensitivity analyses that examined the effect among only those who viewed the films.

**Conclusion:**

The online documentary film (4Weeks2Freedom) designed to boost motivation and self-efficacy and to promote ex-smoker identity does not appear to prompt quit attempts among smokers.

**Implications:**

This trial found that an online documentary film (4Weeks2Freedom) designed to boost motivation and self-efficacy and to promote ex-smoker identity was ineffective in prompting quit attempts among an unselected panel of smokers from the UK. This approach appears unpromising as a means of raising the rate at which smokers attempt to quit and is a low priority for future research.

## Introduction

Tobacco smoking causes more than 6 million deaths across the world each year.^1^ In order to increase the numbers of smokers stopping, it is necessary for smokers to make more attempts to quit and to increase their odds of succeeding. Research into smoking cessation has tended to focus on increasing the odds of success by developing effective smoking cessation treatments but has “relatively” neglected how to encourage more smokers to make an attempt or try more frequently.^2^ Brief advice and motivational interviewing demonstrate the potential for interventions to increase quitting among unselected smokers (ie, not first selected to have a minimum motivation to quit at some point in the future).^3–5^ A recent meta-analysis indicated that smokers receiving brief advice from a physician compared with nothing were about 70% more likely to quit smoking in the long-term as a result of increasing the incidence of attempts.^5^ The majority of smokers included in the analysis were unselected. More intense advice—including more time, materials and follow-up—appears to be less effective for unselected compared with motivated smokers.^5^ Instead, the content of the brief advice is more important: physicians can promote more attempts by offering assistance to all unselected smokers than by advising all to quit and offering assistance only to those who express an interest in doing so.^3^ Meta-analysis also indicates that smokers receiving motivational interviewing are about 30% more likely to quit and that the effect is similar regardless of whether smokers are unselected.^4^

Videos can also promote quitting among smokers.^6–9^ Traditionally, longer videos—or films—have been used to provide individual support to smokers trying to quit^6,9^ or much shorter video clips have been used as part of mass media campaigns to promote attempts.^7,8^ The expansion of digital access has provided the opportunity to deliver motivational video clips or films to smokers at relatively low cost.^10,11^ For example, films can be hosted on dedicated websites or unselected smokers can be exposed to video clips incidentally on social media or during pop-up adverts while watching other online content. These channels are likely to become increasingly important where catch-up television and digital recording reduce the opportunities for exposure to traditional television advertising. It has already been shown that online films to support smokers trying to quit can be effective in improving cessation outcomes: in a large randomized trial smokers willing to quit within 6 months who were assigned to receive films of adults offering tailored cessation advice on a website were more likely to report abstinence 6 months later than those assigned to receive generic text-based cessation advice on a website.^6,11^ It is therefore important to evaluate whether online films can be effective at promoting quit attempts among unselected smokers.

Mass media campaigns usually rely heavily on brief video clips and appear effective in reducing smoking behavior at a population level as part of comprehensive tobacco control programmes.^7,8,12,13^ A review of comparisons of different message types suggested that campaigns focusing on negative health effects and featuring testimonials perform better than those without this content.^14^ However, the comparators have tended to involve anti-industry or how-to-quit themes and until recently there was relatively little research on the effectiveness of campaigns focusing on positive messaging.^14^ A 2012 Stoptober campaign centered around a positive mass quitting trigger was found to be extremely cost-effective^15^ and a recent analysis of all televised tobacco control mass media content in England between 2005 to 2010 indicated that positive campaigns were more effective than negative at increasing quitline calls, after adjusting for seasonal trends, cigarette prices and other tobacco control policies,^16^ while a similar analysis found that per capita exposure to both positive and negative campaigns were associated with declines in smoking prevalence.^17^

4Weeks2Freedom is an online film that was developed to promote quit attempts in the general population of smokers. On the basis of the latest evidence on the effectiveness of different mass media message-types, the film featured positive testimonials from smokers recorded during the first month of successful attempts to quit smoking. The selection of content was informed by the PRIME theory of motivation.^18^ PRIME Theory has many components but in this case the one that was considered most relevant was the idea that one could create a momentary desire and therefore intention to stop smoking by creating a vivid positive image of what it would be like in a way that smokers could identify with. If identification with smokers attempting to quit is successful, it may boost desire to quit both in terms of creating a positive image and also boosting self-confidence in success. These constructs were operationalized as video diaries of smokers who were going through the process with the knowledge that they met their challenge of stopping for 4 weeks as a springboard to lasting cessation.

Thus the current study addressed the question of whether a novel online motivational film designed to boost motivation and self-efficacy and provide role-models to promote ex-smoker identities was effective in promoting quit attempts in the general population of smokers.

## Methods

### Study Design

The study was a three-arm controlled trial between February and March 2014 with participants individually randomized to a no-intervention control, an informational control film, or 4Weeks2Freedom. Assessment was performed at baseline (immediately before allocation to one of the three conditions), and at the endpoint 4 weeks after enrolment. The study was approved by the ethics committee of University College London (CEHP/2013/508).

### Intervention

The intervention was a film called 4Weeks2Freedom that documented the intimate experiences of five smokers during the first month of their successful attempts to quit smoking. The intervention was based on the PRIME theory of motivation and the behavior change techniques of boosting motivation and self-efficacy and promoting an ex-smoker identity.^19^ To provide authentic content eight smokers (of whom five managed a month of abstinence) were each given a camera and asked to record their own video diaries that would provide insight into the experiences of quitting and inspiration for other smokers. The smokers were selected to include a range of different ages, sexes, ethnicities, and socioeconomic status backgrounds. The testimonial content recorded by the five successful smokers was edited to provide material judged by the production team to be likely to boost motivation and self-efficacy by providing positive role modeling that promoted an ex-smoker identity. The video diaries were accompanied by video analysis from R. West with evidence-based advice on stopping. The aim of this advice was to encourage those who made a quit attempt to use support that would give the highest probability of success. Early versions of the film were refined on the basis of focus-group testing with a diverse group of smokers. The final film consisting of both the diaries and the analysis was 90 minutes and all available on a website together that could be viewed in one sitting or as individual chapters over 4 weeks to coincide with the progress of an attempt. Smokers in the focus groups found this concept acceptable. The film was hosted on a website using an identical style and layout to that used for the presentation of the informational control film. The film and website are both freely available to view: www.4weeks2freedom.com/.

### Control

The informational control film featured a cessation expert (R. West) discussing the health risks of smoking and the reasons that people smoke. The film and website on which it was presented are both freely available to view: www.smokefreeadvice.com/. The no-intervention control condition received no information after initial enrolment. After completing the 4-week study endpoint outcomes, both control groups were provided with a link to the website hosting 4Weeks2Freedom.

### Procedure, Randomization, and Masking

As members of an online panel maintained by Ipsos MORI, participants were invited by email to complete an online survey and possibly view films about smoking. Respondents were told that by completing the survey they would earn points which could be redeemed against high street vouchers or used to enter a prize draw. Those interested in participating after reading the study information and eligibility criteria were asked for consent and to complete the baseline questionnaire. Those completing the questionnaire were enrolled and randomly allocated in a 1:1:1 ratio to one of the three conditions. This involved seeing a webpage thanking them for their participation and a reminder of the follow-up in 4 weeks, and containing (1) no more information (no-intervention control), (2) a link to the website hosting the informational control film, or (3) a link to the website hosting the intervention film. The links to the films were qualified by an explanation that a research team at University College London had produced a video about stopping smoking and would appreciate their reaction, and finally requested “please click on the link to have a look even if only for a few minutes.” Each group also received an email sent almost immediately after they completed the survey that reiterated the information provided on each of their respective final webpages. The random treatment allocation was automated with no experimenter involvement by use of an unseen random number function embedded in the website code and concealed from participants until they were included in the study. The randomization was at the individual level with no restriction (ie, no blocking). Once allocated to a condition, the email address of each participant was secured to that allocation to prevent contamination. The endpoint follow-up was 4 weeks after enrolment. Follow-up data were automatically collected via an online questionnaire emailed to participants. Nonresponders were sent up to three email reminders.

### Study Sample

Participants were adults (aged 18 and over) from the United Kingdom who smoked cigarettes (including hand-rolled) daily or occasionally at the time of the survey and who were willing to: view films about smoking, be followed up at 4 weeks, and provide informed consent.

### Measures

All variables listed in Table 1 were assessed at baseline. Measures recorded for outcome assessment at the 4-week endpoint were: self-report of a serious attempt to quit smoking permanently in the previous 4 weeks and, among those who attempted to stop, whether nonsmoking was continued since the start of the attempt to the time of the survey, and which (if any) smoking cessation aids were used (see list in Supplementary Materials). Additionally, those allocated to either the informational control film or 4Weeks2Freedom condition were asked whether they had viewed the film, and those who reported having seen it were asked to indicate their satisfaction with their respective films on four dimensions: participants were asked to provide “yes” or “no” responses on whether they (1) found it to be helpful, (2) personally relevant, (3) would recommend it to others, and (4) use it in the future.

**Table 1.** Characteristics of Participants on Entry to the Study

Characteristic	No-intervention control (*N* = 1016)	Informational control film (*N* = 1004)	4Weeks2Freedom (*N* = 999)	Total (*N* = 3019)
% (*N*) Female	47.3 (481)	48.9 (491)	48.0 (480)	48.1 (1452)
Mean (*SD*) age in years	42.9 (14.4)	42.4 (14.2)	43.0 (14.7)	42.7 (14.4)
% (*N*) Married	53.1 (540)	57.0 (572)	51.0 (509)	53.7 (1621)
% (*N*) Having children	55.3 (562)	54.1 (543)	52.6 (525)	54.0 (1630)
% (*N*) White ethnicity	92.3 (938)	91.2 (916)	92.3 (922)	92.0 (2276)
% (*N*) Currently in full-time education	4.7 (48)	5.9 (59)	6.8 (69)	5.8 (175)
% (*N*) No post-16 years old educational qualification	34.1 (346)	33.2 (333)	36.3 (363)	34.5 (1042)
% (*N*) Lower socioeconomic status^a^	51.8 (526)	48.7 (489)	51.9 (518)	50.8 (1533)
Mean (*SD*) cigarettes per day smoked	13.2 (9.5)	12.5 (8.8)	13.4 (9.5)	13.0 (9.3)
% Nondaily smokers	18.1 (184)	20.1 (202)	18.4 (184)	18.9 (570)
Mean (*SD*) age of smoking initiation	17.1 (4.3)	17.3 (4.8)	17.4 (4.9)	17.3 (4.6)
% (*N*) no support in past year quit attempt	52.9 (537)	52.0 (522)	53.3 (532)	52.7 (1591)
% (*N*) no behavioral support in past year quit attempt	91.5 (930)	89.2 (896)	91.2 (911)	90.7 (2737)
% (*N*) Made quit attempt in the previous year	31.7 (322)	32.8 (329)	31.5 (300)	31.5 (951)
% (*N*) Do not want to stop smoking^b^	29.6 (301)	30.5 (306)	32.7 (327)	30.9 (934)
% (*N*) Never stopped for more than a week	43.3 (440)	42.3 (425)	44.3 (443)	43.3 (1308)
% (*N*) Usually smokes within 5 minutes of waking	19.4 (197)	18.1 (182)	21.8 (218)	19.8 (597)
Mean (*SD*) FTND score (0–10)^c^	3.6 (2.6)	3.5 (2.5)	3.8 (2.5)	3.7 (2.5)
Mean (*SD*) Time with smoking urges score (0–5)	2.1 (1.1)	2.0 (1.1)	2.1 (1.1)	2.1 (1.1)
Mean (*SD*) Strength of smoking urges score (0–5)	2.2 (1.0)	2.1 (1.0)	2.2 (1.0)	2.2 (1.0)
Mean (*SD*) MPSS-mood subscale (0–4)^d^	1.0 (0.9)	1.0 (0.8)	1.1 (0.8)	1.0 (0.8)

MPSS = Mood and Physical Symptoms Scale; MTSS = Motivation to Stop Scale.

^a^Consisted of individuals who have never worked, were long term unemployed or were from routine and manual occupations according to the National Statistics Socio-Economic Classification self-coded method.^20^

^b^This is the proportion responding “I don’t want to stop smoking” or “I think I should stop smoking but don’t really want to” on the MTSS.^21^

^c^Fagerström test for nicotine dependence.^22^

^d^The mood and physical symptoms scale is the mean of responses to five separate: depressed, irritable, restless, hungry, and poor concentration.

The primary outcome was a serious attempt to stop smoking permanently in the 4 weeks between assignment and the study endpoint. The secondary quitting outcome was self-reported nonsmoking following a serious attempt to stop smoking permanently at the 4-week study endpoint. On the basis of the intention-to-treat principle, those who failed to respond to endpoint follow-up attempts were retained in the analyses and classified as having not made an attempt to stop. The remaining secondary outcomes were use of smoking cessation treatments and satisfaction ratings.

### Sample Size

The sample size was determined with alpha set at 5% and to provide 80% power to detect a projected 3.5% intervention difference compared with the no-intervention control (ie, 10% vs. 6.5%). The anticipated effect size is based on other interventions that have attempted to increase quit attempts in the general population^15,23^ and what is known about the rate of past-month quit attempts in England.^15^ By basing the effect size on real-world interventions and their effect on the general population, the power calculation allowed for a proportion of the participants having minimal exposure to the intervention. Hence, a minimum total sample size of 1947 was required in the intervention group and no-intervention control group, and therefore recruitment was specified to continue until 3000 participants had been recruited across the three arms of the trial. We assumed that there would be approximately 20% attrition on the basis of a recent online trial,^24^ but made no adjustment to the target sample size. Instead, those who failed to respond to endpoint follow-up attempts were retained in the primary analysis and classified as having not made an attempt to stop. We did not alter the estimated effect size because we assumed the vast majority of those who did not respond would not have made an attempt.

### Analysis

Univariable logistic regression models were used to analyze the dichotomous primary and secondary quit attempt and smoking cessation outcomes. We regressed each outcome in a separate model onto treatment allocation (reference: 4Weeks2Freedom). The associated 95% confidence intervals were calculated. As sensitivity analyses to adjust for any chance imbalances in baseline characteristics, we also constructed a multivariable logistic regression model for each of the dichotomous outcomes including all variables listed in Table 1. In an additional sensitivity analysis, the models comparing the informational control and 4Weeks2Freedom film were reexamined among only those participants (1) who were recorded clicking the link when it was first presented to them following the baseline survey and (2) who at the study endpoint reported having viewed the film at any stage in the previous 4 weeks. In a post hoc analysis suggested by reviewers, we compared the primary outcome for those assigned to the no-intervention control with those participants who reported having viewed (1) the 4Weeks2Freedom film and (2) the informational control film.

The Neyman–Pearson approach to statistics (of which the analyses described above are an example) focuses on the probability of data given the null hypothesis and provides a reliable decision procedure with controlled long-term error rates.^25^ A limitation is that in the event of “nonsignificant” results, that is, failing to reject the null hypothesis, this approach is unable to distinguish between insensitive data and the null hypothesis being correct.^25^ The calculation of a Bayes factor establishes the relative likelihood of the null versus the experimental hypothesis. A Bayes factor (*B*) represents the strength of support for the alternative hypothesis (H1) relative to the null,^25^ with *B* > 1 indicating support for H1 and *B* < 1 indicating support for the null. Values of *B* greater than 3 or smaller than 1/3 are typically regarded as providing substantial evidence in favor of H1 or the null hypothesis, respectively, while intermediate values indicate the data are insensitive.^26,27^ Bayes factors were calculated for each analysis described above separately with the alternative hypotheses conservatively represented in each case by a half-normal distribution see for the online calculator used: www.lifesci.sussex.ac.uk/home/Zoltan_Dienes/inference/Bayes.htm.^25^ In an alternative hypothesis represented by a half-normal distribution, the standard deviation of a distribution can be specified as an expected effect size, which means plausible values have been effectively represented between zero and twice the effect size, with smaller values more likely. The expected effect size used for each comparison is provided below Table 2.

**Table 2.** Effect of 4Weeks2Freedom on Quitting

	1. No-intervention control	2. Informational control film	3. 4Weeks2Freedom	3. vs. 1.	3. vs. 2.
				*OR* (95% CI), Bayes factor	*OR* (95% CI), Bayes factor
	Percent (numbers)				
Primary outcome: quit attempt					
Full sample	24.5 (249/1016)	24.6 (247/1004)	24.3 (243/999)	0.99 (0.81 to 1.21), 0.20^a^	0.99 (0.80 to 1.21), 0.27^b^
Subsample: clicked link in first session	—	26.1 (86/329)	23.6 (91/386)	—	0.87 (0.62 to 1.23), 0.21^c^
Subsample: reported viewing the film	—	37.0 (206/557)	37.5 (211/563)	—	1.02 (0.80 to 1.30), 0.29^d^
Secondary outcome: self-reported nonsmoking					
Full sample	7.5 (76/1016)	6.1 (61/1004)	6.8 (68/999)	0.90 (0.64 to 1.27), 0.39^e^	1.13 (0.79 to 1.61), 1.07^f^
Subsample: clicked link in first session	—	5.2 (17/329)	5.2 (20/386)	—	1.00 (0.52 to 1.95), 0.8^g^
Subsample: reported viewing the film	—	9.0 (50/557)	10.7 (60/563)	—	1.21 (0.81 to 1.80), 1.25^h^

CI = confidence interval; *OR* = odds ratio. The primary outcome was a serious attempt to stop smoking permanently in the past 4 weeks. The secondary outcome was self-reported nonsmoking at the 4-week endpoint following a serious attempt to stop. Those lost to follow-up were counted as treatment failures. The primary analyses were all unadjusted but adjusted analyses were performed as a sensitivity analysis and had no effect on the pattern of results. The adjusted models included all characteristics presented in Table 1. For the Bayesian analyses, the effect sizes used to specify the *SD* for the half-normal distributions representing the alternative hypotheses were as follows: ^a^ *OR* = 1.6; ^b^ *OR* = 1.4; ^c^ *OR* = 1.6; ^d^ *OR* = 1.6; ^e^ *OR* = 1.3; ^f^ *OR* = 1.2; ^g^ *OR* = 1.3; ^h^ *OR* = 1.3. In sensitivity analyses marginally varying the magnitude of these effect sizes, the pattern of results was similar.

## Results

### Characteristics of the Participants

**Figure 1.**
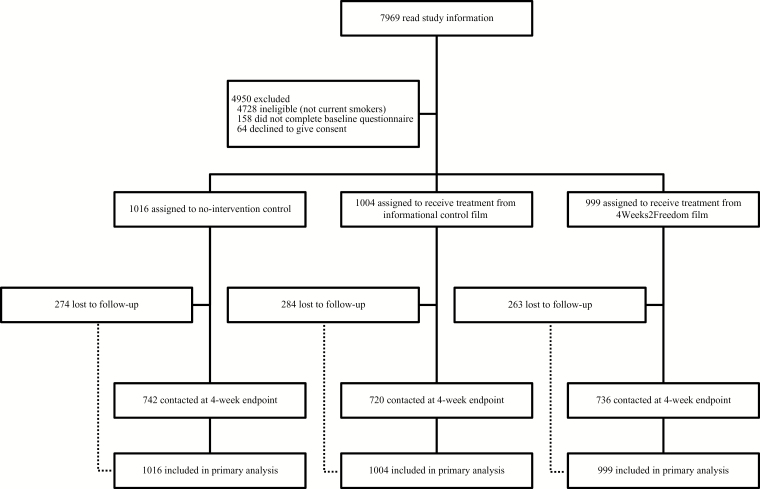
Numbers of participants enrolled in the study and included in the primary analysis.

Figure 1 shows the flow of participants through the trial.^28^ Of 7969 people from an online panel who read study information, 3019 were enrolled and randomized (no-intervention control *n* = 1016; informational control film, *n* = 1004; 4Weeks2Freedom, *n* = 999). The most common reason for exclusion was being ineligible (not being a current smoker, *n* = 4728) while a small number of current smokers were excluded because they did not complete the baseline questionnaire (*n* = 158) or declined to give consent (*n* = 64). The first participant was enrolled on February 19, 2014, and the last follow-up contact was on April 07, 2014.

Of 3019 randomized, 821 (27.2%) did not respond at the endpoint and were assumed to have not made a quit attempt. This rate was similar between the three groups (27.0%, 28.3%, and 26.3%) and the baseline characteristics of those lost to follow-up did not differ significantly between the groups (data not shown).

In the recruited sample, the mean age and proportion of women were similar to the smoking population in England but the proportion with lower socioeconomic status was smaller, which may have reflected the online recruitment because smokers with lower socioeconomic status tend to have less internet access (Table 1).^29,30^ The daily cigarettes smoked and measures of tobacco dependence were broadly representative of the general population.^22^ Approximately a third had made an unsuccessful attempt to stop in the past year and a third also reported not currently wanting to stop smoking. Baseline characteristics were similar between the three groups.

### Outcomes

No effect of the intervention on the primary or secondary outcome was detected when compared with either the no-intervention control or the informational control film (Table 2). Adjustment for all baseline characteristics shown in Table 1 had a negligible effect on these comparisons. In total, 32.7% (329/1004) of participants clicked the link to view the informational control film immediately after the baseline questionnaire, and 38.6% (386/999) clicked immediately to 4Weeks2Freedom. At the follow-up, 55.5% (557/1004) reported viewing the informational control film, and 56.4% (563/999) 4Weeks2Freedom. In sensitivity analyses, the pattern of results was unchanged in analyses repeated among only (1) those participants who were recorded immediately clicking the link and (2) those who reported having viewed the film. In a post hoc analysis, we found that those who reported having viewed the 4Weeks2Freedom film were more likely to have attempted to quit smoking than those assigned to the no-intervention control (odds ratio [*OR*] = 1.85, 95% CI = 1.48 to 2.31). However, the effect on quit attempts was similar for those who reported having viewed the informational control film compared with the no-intervention control (*OR* = 1.81, 95% CI = 1.45 to 2.26).

The calculation of Bayes factors indicated that the evidence supported the hypothesis of the intervention being no more effective at promoting quit attempts than either the no-intervention control or the informational film (Table 2). Bayes factors derived from the cessation outcomes indicated that these data were insensitive but tended to support the hypothesis that 4Weeks2Freedom was ineffective.

Table 3 shows that among those who reported viewing their respective films, the level of satisfaction was similar between the groups receiving the two different films. The usage of treatment among those who attempted to quit smoking was similar between the three groups, with e-cigarettes being the most commonly reported aid to cessation (Supplementary Materials).

**Table 3.** Satisfaction With the Treatment Films

	2. Informational control film (*n* = 557)	3. 4Weeks2Freedom (*n* = 563)	*OR* (95% CI)
	Percent (numbers)		
Helpfulness	64.8 (361)	62.2 (350)	0.89 (0.70 to 1.14)
Personal relevance	60.3 (336)	58.4 (329)	0.92 (0.73 to 1.17)
Recommendation	71.3 (397)	71.8 (404)	1.02 (0.79 to 1.33)
Use in future	61.6 (343)	64.3 (362)	1.12 (0.88 to 1.43)

CI = confidence interval; *OR* = odds ratio. Of the 1456 who responded and were in the control or 4Weeks2Freedom conditions, 336 reported that they did not view their respective films and therefore could not provide data for these analyses. All analyses in this table are unadjusted.

## Discussion

This trial found that 4Weeks2Freedom—a documentary film designed to boost motivation and self-efficacy and to promote ex-smoker identity—was ineffective compared with a no-intervention control or informational control film in prompting quit attempts in an unselected sample of smokers from the United Kingdom. The finding that quit attempts were similar among the subsamples of those who reported viewing the intervention and informational control but both were greater than among those allocated to no control suggests both may have had some effect and the issue may have been more specifically with the intervention content compared with the informational control film. However, this finding needs to be regarded with caution as it is not possible to rule out self-selection, that is, that the difference in quit attempts resulted from those who are more likely to click a link being more likely to quit, than being attributable to either film exposure increasing the propensity of a smoker to make a quit attempt.

There are several possible reasons why the film was ineffective in promoting quit attempts. First of all, the film may not have been sufficiently engaging to keep participants viewing for long enough to have an effect. We do not have data on how long the films were viewed, but it is noteworthy that there was no effect in smokers who clicked on the link immediately who may have been expected to be view the films for longer. A second explanation is that the intervention was not successful in getting viewers to identify with the people in the film. We do not have information on this. We decided to minimize the response burden on participants to maximize response rate. However, it would be possible to show the film to another sample to gauge level of identification with smokers in the film. It is also possible that viewers did not consider the experience of the smokers in the film to be positive, which is supported by the similar ratings of satisfaction given to the intervention and control films among those who viewed them. Finally, despite the overall concept being received positively in focus groups, it is possible that the general approach of extended documentary films to motivate cessation is likely to have limited success because a major barrier to quitting is not lack of identification with quitters or lack of confidence in quitting but rather that smoking is enjoyable.^31,32^ Notwithstanding the limitations of the current study, given that we know that brief mass media messages—albeit with repeated exposure—can be effective at promoting quitting among unselected smokers,^7,8,12,14^ as can brief advice from physicians and motivational interviewing,^3–5^ it may be that extended films that require a high level of engagement to be effective are not an appropriate strategy and a low priority for future research.

A strength of this study is the calculation of Bayes factors. Although many studies reporting null results interpret their failure to reject a null hypothesis as evidence of no difference, the correct interpretation is that such findings provide no evidence of a difference.^25,26^ By contrast, the Bayes factors calculated in the current study show that the data support the conclusion that the intervention was ineffective, insofar that one believes that an effective intervention would be plausibly modeled by a half-normal distribution with a standard deviation relating to the specified expected effect sizes (the half-normal is quite conservative as it means plausible values have been effectively represented between zero and twice the expected effect, with smaller values more likely). However, it is possible due to the low compliance—only half reported viewing the film and only a third immediately clicked on the link to the film—and the assumption that nonresponders had not made an attempt, that other researchers may believe an effective intervention in this context would have been more suitably represented by an expected effect size of *OR* = 1.3 (rather than *OR* = 1.6) compared with the no-intervention control and *OR* = 1.2 (rather than *OR* = 1.4) compared with the informational control film, in which case they may regard the results as somewhat insensitive. The fact that compliance was low is a critical limitation of this study and creates more scope for legitimate disagreement about the meaning of the results than would be ideal.

This research had other important limitations. Participants were recruited by means of a small financial incentive (points with financial value). This may have created an extrinsic motivation to take part in the study but not necessarily to engage with the film. It is possible that were the film to be offered in a different context, for example, recommended by a health professional to unselected smokers, then a different result would have been obtained. Another limitation is that no data were collected on mediating variables that may have helped to explain the findings. This was because we wished to prioritize engagement with the primary outcome measure, and minimize mere-measurement effects that would limit generalizability.^33–35^ The result, however, is that we do not know whether the intervention failed to boost motivation and self-efficacy, and create the kind of identification with those ex-smokers portrayed in the film that was hoped for or whether such identification, or improved motivation and self-efficacy, were insufficient to prompt a quit attempt. It may have been possible to distinguish these possibilities if we had nested a process evaluation within the trial including a qualitative exploration of the views and experiences of a subset of users. Future research should follow best practice recommendations to include such work.^36^

There remains the question as to whether a film of this kind might be helpful in supporting smokers who are already engaged in a quit attempt. Showing clips of smokers experiencing similar emotional and physical reactions to abstinence and how they deal with it could be motivational and supportive. Such clips could, for example, be included in an online intervention or a smartphone application for smokers wanting help with stopping.^6,9^ The film could also be useful as a training tool for stop-smoking practitioners in showing them what smokers are experiencing.

In conclusion, this study found an online film designed to boost motivation and self-efficacy and to promote ex-smoker identity by showing video diaries of smokers quitting to be ineffective in prompting quit attempts in an online panel of smokers. This approach appears unpromising as a means of raising the rate at which smokers attempt to quit. It remains to be seen whether it could help in other ways, for example, as part of an intervention to support smokers who are already trying to quit.

## Supplementary Material

Supplementary Materials can be found online at http://www.ntr.oxfordjournals.org

## Funding

This work was supported by Cancer Research UK (C1417/A7972) who provided funding for the conduct of this research and preparation of the manuscript. JB’s post is funded by a fellowship from the UK Society for the Study of Addiction; RW is funded by Cancer Research UK. Funding was provided for the conduct of this research and preparation of the manuscript. The funders had no final role in the study design; in the collection, analysis and interpretation of data; in the writing of the report; or in the decision to submit the article for publication. All researchers listed as authors are independent from the funders and all final decisions about the research were taken without constraint by the investigators.

## Declaration of Interests

*JB has received an unrestricted research grant from Pfizer. RW undertakes research and consultancy and receives fees for speaking from companies that develop and manufacture smoking cessation medications (Pfizer, J&J, McNeil, GSK, Nabi, Novartis, and Sanofi-Aventis). RW co-owns the commercial rights to 4Weeks2Freedom. SM and MW have no conflicts.*

## Supplementary Material

Supplementary Data
