# Supplementary material for: An Online Documentary Film to Motivate Quit Attempts Among Smokers in the General Population (4Weeks2Freedom): A Randomized Controlled Trial
Source: Nicotine Tob Res. 2015 Jul 27;18(5):1093–100. doi: 10.1093/ntr/ntv161 (PMC4826487; doi:10.1093/ntr/ntv161)
Supplement: Supplementary Data [file supp_18_5_1093__index.html]

An online documentary film to motivate quit attempts among smokers in the general population (4Weeks2Freedom): A randomised controlled trial — An Online Documentary Film to Motivate Quit Attempts Among Smokers in the General Population (4Weeks2Freedom): A Randomized Controlled Trial — An Online Documentary Film to Motivate Quit Attempts Among Smokers in the General Population (4Weeks2Freedom): A Randomized Controlled Trial — Supplementary Data 

# An Online Documentary Film to Motivate Quit Attempts Among Smokers in the General Population (4Weeks2Freedom): A Randomized Controlled Trial

## Supplementary Data

Data files

- Supplementary Data - Supplementary Data
