# Supplementary material for: An Online Documentary Film to Motivate Quit Attempts Among Smokers in the General Population (4Weeks2Freedom): A Randomized Controlled Trial
Source: Nicotine Tob Res. 2015 Jul 27;18(5):1093–100. doi: 10.1093/ntr/ntv161 (PMC4826487; doi:10.1093/ntr/ntv161)
Supplement: Supplementary Data [file supp_ntv161_Supp_materials_2.docx]

# Table S1 –Usage of other treatments

|  | **1. No-intervention**  **(n=249)** | **2. Informational control film**  **(n=247)** | **3. 4Weeks2Freedom**  **(n=243)** |
| --- | --- | --- | --- |
|  | **Percent (numbers)** | | |
| Face-to-face behavioural support | 8.4 (21) | 4.5 (11) | 7.0 (17) |
| Other behavioural support† | 21.7 (54) | 23.5 (58) | 20.6 (50) |
| Nicotine replacement therapy: prescription | 12.9 (32) | 14.6 (36) | 15.2 (37) |
| Nicotine replacement therapy: over-the-counter | 34.5 (86) | 26.3 (65) | 34.6 (84) |
| Varenicline | 3.2 (8) | 6.9 (17) | 2.9 (7) |
| Zyban | 4.8 (12) | 2.8 (7) | 3.3 (8) |
| E-cigarettes | 37.8 (94) | 37.2 (92) | 41.2 (100) |

† Other includes support delivered by telephone, booklet, website, or ‘app’.
